# Influence of Ultraviolet Radiation Exposure Time on Styrene-Ethylene-Butadiene-Styrene (SEBS) Copolymer

**DOI:** 10.3390/polym12040862

**Published:** 2020-04-09

**Authors:** Daniel Garcia-Garcia, José Enrique Crespo-Amorós, Francisco Parres, María Dolores Samper

**Affiliations:** Materials Science Division, Technological Institute of Materials, Universitat Politècnica de València, 03801 Alcoy, Alicante, Spain; dagarga4@epsa.upv.es (D.G.-G.); jocream@dimm.upv.es (J.E.C.-A.); fraparga@dimm.upv.es (F.P.)

**Keywords:** surface modification, degradation, AFM, mechanical properties, block copolymer

## Abstract

The effect of ultraviolet radiation on styrene-ethylene-butadiene-styrene (SEBS) has been studied at different exposures times in order to obtain a better understanding of the mechanism of ageing. The polymer materials were mechanically tested and then their surfaces were analyzed using a scanning electron microscope (SEM) and atomic force microscopy (AFM). Moreover, the optical analysis of contact angle (OCA) was used to evaluate the surface energy (γ_s_) and the yellowing index (YI) and attenuated total reflectance infrared spectroscopy (ATR–FTIR) were used to observe structural and physical changes in aging SEBS. The results obtained for the SEBS, in relation to the duration of exposure, showed superficial changes that cause a decrease in the surface energy (γ_s_) and, therefore, a decrease in surface roughness. This led to a reduction in mechanical performance, decreasing the tensile strength by about 50% for exposure times of around 200 hours.

## 1. Introduction

Styrene-ethylene-butadiene-styrene (SEBS) is considered a thermoplastic elastomer (TPE); these materials combine the easy processing characteristic of a thermoplastic with the physical properties of a vulcanized rubber [1,2]. SEBS is obtained by the hydrogenation process of styrene-butadiene-styrene (SBS) that breaks the unsaturated bonds of the polybutadiene chain, and the result is a transparent material with a higher resistance to aging that is used in areas such as medical instruments, orthopedic appliances, toys, protective materials, polymer modified bitumen (PMB), and so on [3,4].

TPEs, like organic polymers, degrade under environmental conditions owing to solar radiation and the presence of oxygen [5]. However, it should be taken into account that the type of degradation will depend on different factors such as environmental conditions, the production method, and the structure of the polymer [6,7]. TPEs are relatively new materials and their applications are limited, so there is only a small amount of research about their degradation. The TPEs’ degradation can be generated in the polystyrene phase or in the elastomer phase [8], although this latter case is the one that most often undergoes degradation owing to its low *T*_g_; in addition, the block copolymers are highly permeable to oxygen. Therefore, the composition of the central block will determine the thermal behavior of the styrene elastomers during ageing [9,10]. Moreover, aging also depends on the butadiene/styrene ratio and block structure, owing to radial block structures having faster aging than linear block structures, although this behavior can be slowed down using carbon nanotubes in SBS copolymer [11]. Tomacheski et al. [12] studied the use of antimicrobial metal such as silver to protect TPE against microbial attack. However, after nine months of weathering exposure, the compounds lost their antibacterial properties; therefore, the incorporation of silver-based additives in TPE does not guarantee better mechanical resistance after exposure weatherproof.

The presence of the polydiene phase in the unsaturated styrene elastomers only gives a limited resistance to ageing as the elastomeric segments contain a double bond for each monomer unit. These links are very reactive and they limit the stability of the product when it is exposed to high temperatures, humidity, UV radiation, or ozone [13,14]. However, hydrogenated elastomers such as poly[*b*-ethylene-*co*-butylene-*b*-styrene] triblock copolymer (SEBS) are saturated, and are thus much more stable [11].

A previous study into the photo-oxidative degradation of SEBS [15,16] showed changes in the phase partitioning of the polystyrene olefins, with the formation of acetone groups in the styrene units and carboxylic acids in the olefin end chains. Another aspect to take into account once the material is degraded or aged is its surface morphology, where surface energies and adhesion phenomena are involved.

Some TPEs are used in bitumen modification and then they are used in the preparation of modificated asphalts, which are used in special pavements to suppress the noise level or to drain out water readily, among other desirable effects. However, owing to the sensitivity of TPEs, which tend to be degraded by exposure to heat and UV light, these conditions usually occur during storage, mixing, transport, and laying, as well as in service life. In order to reduce the ageing, some researchers work in different studies to improve the aging resistance of SBS modified bitumens using antioxidants like Irganox 1010 and Irgafos 168, both extracted by acetone, to reduce the thermal oxidation [17]. On the other hand, Xu X. et al. [18] used the synergistic effect of 4,4’-diphenylmethane diisocyanate and 1,4-butanodiol diglycidyl ether in rejuvenation of aged SBS. However, SEBS modified bitumens reduce the ageing effect on physical and rheological properties compared with SBS modified bitumen [19], but it had to be remembered that the properties of modified bitumen are largely influenced by the process of ageing.

It is widely recognized in the aerospace and automobile industries that adhesion mechanisms are dependent on the surface characteristics of the material in question. Over the last 30 years, understanding of adhesion mechanisms has increased significantly in both sectors as these materials are as lighter, cheaper alternatives to metals and metal components [20,21,22,23,24,25].

Describing adhesion mechanisms in simple terms is difficult owing to the complexity and still developing understanding of the subject [26,27]. The ultimate goal is to identify a single mechanism that explains all adhesion phenomena [28,29,30].

Any consideration of adhesion mechanisms requires information regarding the physical and chemical properties of surface adhesion, or separation in the case of surfaces where adhesion has failed during use or as a result of mechanical testing [31,32,33,34]. There are a number of surface characterization techniques that can be used to investigate adhesion mechanisms and resistance to adhesion. These include secondary ion mass spectroscopy (TOF-SIMS), X-ray photoelectron spectroscopy (XPS), atomic force microscopy (AFM), scanning electron microscopy (SEM), attenuated total reflection infrared spectroscopy (ATR-IR), and other microscopy techniques more sensitive to surface energy such as the optical analysis of contact angle (OCA). Numerous studies have used these techniques to analyze surface properties such as roughness, polarity, chemical composition, and surface free energy in order to describe and explain the adhesion phenomena of a surface or interface [35,36,37,38,39,40,41].

This work aims to analyze the effects and their impact on SEBS when it is subjected to accelerated ageing in a UV chamber. These effects and their impacts on the material are observed and quantified by a number of techniques, such as structural and morphological analysis in order to test the behavior observed in the aged material.

## 2. Materials and Methods

### 2.1. Materials and Sample Preparation

The styrene-ethylene-butadiene-styrene (SEBS) block copolymer used was a commercial grade material Megol DP 1261/s Cristallo E251, Applicazioni plastiche industriali S.p.A. (Mussolente, Italy).

Specimens were prepared by injection moulding using a conventional injection moulding machine Meteor 270/75 (Mateu & Sole, Barcelona, Spain) with a mold with normalized sample dimensions for tensile test according to ISO-527-2, specifically 1A samples. This machine has four heating zones and the temperatures from the feeding zone to the die zone were set at 170, 175, 180, and 185 °C, respectively.

### 2.2. Exposure Conditions

The samples were subjected to aging solar ultraviolet (UV) accelerated in a xenon chamber XENOTERM RA-1500 (ICC, Barcelona, Spain). The maximum surface area of sun exposure is 300 × 250 mm with a useful height of 300 mm. The samples were placed in the middle of the chamber, with a 150 mm Xenon lamp power 300 W with luminous flux of 22,000 lm, color temperature of 6500 K, and color rendering index of 85.

The adopted references used for the SEBS samples subjected to UV accelerated ageing when carrying out this work can be seen in Table 1.

### 2.3. Mechanical Characterization

The tensile properties of samples were obtained using the universal testing machine ELIB 30 (S.A.E. Iberstest, Madrid, Spain) according to regulation ISO 527. The load cell employed had a capacity of 5 kN and the displacement velocity of the applied clamp was 50 mm/min. Between five and ten test samples were tested at room temperature for each formulation in this study and the results obtained give the mean values. Shore D hardness was measured using a Baxlo durometer (Baxlo, Barcelona, Spain) according to ISO 868. A minimum of five samples were analyzed in order to obtain each result.

### 2.4. Colorimetry

The color measurement was performed with HunterLab reflection spectrophotometer (ColorFlex) using standard light D65 and a 10° standard observation angle. To measure the color of specimen, the detector was simply touched to the surface [15,42]. The tristimulus value *X*, *Y*, and *Z* based on Commission Internationale de l’Eclairage (CIE) standard colorimetric system were measured. The yellowness index (*YI*) based on ASTM E313 was calculated from Function (1) using *X*, *Y*, and *Z*. The Δ*YI* was derived from the *YI* using Function (2). The *YI* of the nondegraded specimen is defined as *YI*_0_. The reported values are the average of five measurements on specimens.

(1) $$YI=\frac{100\left(1.3013X-1.1498Z\right)}{Y}$$

(2) $$\Delta YI=YI-YI_{0}$$

### 2.5. Optical Contact Angle Analysis (OCA)

For the wet surface, EASYDROP standard equipment KRUS brand was used, model FM140 110/220 V, 50/60 Hz. The range of measurements varied between 1° and 180° with an accuracy of ±0.1°. Through the analysis software SW21 DROP SHAPE ANALYSIS (DSA1), ten measurements of each individual drop were taken. The test liquids used for contact angle measurements were selected to include a range of liquids with different polar and dispersive constants and correctly measure changes in surface free energy values. The selected liquids were water, glycerol, diiodomethane, and formamide; Table 2.

The Owens–Wendt method was chosen to calculate surface free energies owing to its simplicity and also because it takes dispersive and polar components into account [43,44,45,46]. The general expression of the Owens–Wend method is as follows:

(3) $$\frac{\gamma_{l}\left(1+\cos\left(\theta \right)\right)}{2{\left(\gamma_{l}^{d}\right)}^{1/2}}={\left(\gamma_{s}^{p}\right)}^{1/2}\cdot \left[\frac{{\left(\gamma_{l}^{p}\right)}^{1/2}}{{\left(\gamma_{l}^{d}\right)}^{1/2}}\right]+{\left(\gamma_{s}^{d}\right)}^{1/2}$$

In this equation, *θ* is the contact angle, $\gamma_{l}$ is the surface tension of the liquid, and γ_s_ is the surface tension of the solid or surface free energy. The terms with the subscripts *d* and *p* refer to the dispersive and polar component, respectively. This expression can be written in terms of a linear equation (y = ax + b); so if we represent ${\left(\gamma_{l}^{p}\right)}^{1/2}/{\left(\gamma_{l}^{d}\right)}^{1/2}$ versus $\gamma_{l}\left(1+\cos\left(\theta \right)\right)/2{\left(\gamma_{l}^{d}\right)}^{1/2}$, we obtain a linear representation. The slope of the line is ${\left(\gamma_{l}^{p}\right)}^{1/2}$, while ${\left(\gamma_{l}^{d}\right)}^{1/2}$ represents the intercept of this line on the *y* axis. Once the polar $\left(\gamma_{s}^{p}\right)$ and dispersive $\left(\gamma_{s}^{d}\right)$ contributions are calculated, the total surface free energy is the sum of these two components $\gamma_{s}=\gamma_{s}^{p}+\gamma_{s}^{d}$.

### 2.6. Attenuated Total Reflectance Infrared Spectroscopy (FTIR–ATR) Surface Analysis

This technique is very useful to obtain the chemical changes induced by the aging treatment [15]. The infrared analysis was performed on a Perkin–Elmer spectrum BX spectrometer (Perkin–Elmer España SL, Madrid, Spain) equipped with attenuated total reflection (ATR) accessory. A total of 150 scans with a resolution of 4 cm^−1^ were carried out for each one of the samples exposed to aging.

### 2.7. Surface Morphology Study

The study of the failure mechanisms was conducted by directly observing the morphology of the fracture surface of the tensile test specimens. Scanning electron microscopy (SEM) was applied in order to analyze the polymer fractures, using an FEI QUANTA 200 (FEI, Hillsboro, OR, USA). The micrographs were obtained in the microscope in environmental mode (ESEM), which does not require the recovery of gold or any other material for a correct visualization. Additionally, the exposure surface was observed by a stereoscopic loupe Olympus SZX7 at 20 magnification, and all samples were coated with a thin layer of graphite with the objective of obtaining images with good contrast.

Atomic force microscopy (AFM) was used to determine the surface topography and roughness of the aged samples [20,47,48]. AFM analysis was performed on a Multimode AFM microscope with a Nanoscope IIIa ADCS controller (Veeco Metrology Group, Cambridge, UK). A monolithic silicon cantilever (NanoWorld Pointprobe^®^ NCH, Neuchâtel, Switzerland) with a force constant of 42 N/m and a resonance frequency of 320 kHz was used to work out the tapping mode. By taking the analysis of the images, the root-mean-squared roughness (Rrms) and the maximum height for the topographic profiles measured on 50 µm × 50 µm images were evaluated.

## 3. Results and Discussion

### 3.1. FTIR–ATR Surface Analysis

Materials were exposed to UV radiation in a xenon chamber before being examined in order to establish the effects of prolonged UV exposure on the properties of the material.

**Figure 1:**
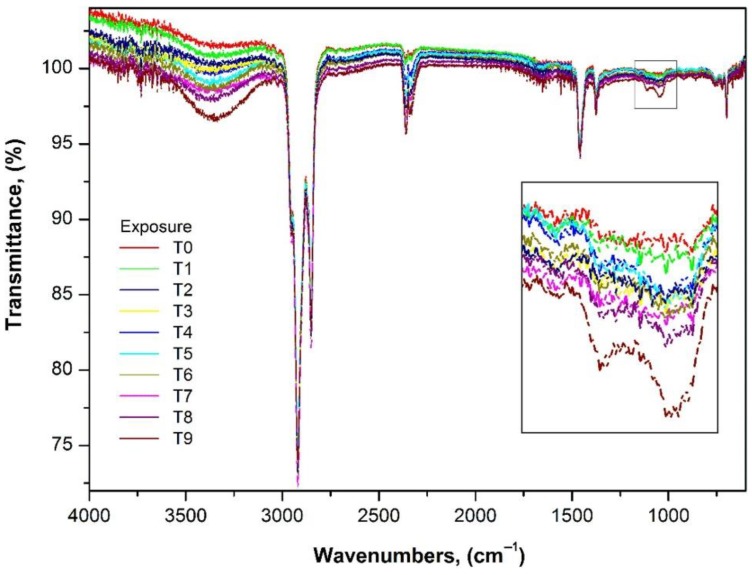
Fourier transform infrared (FTIR) spectra in the range of 4000–400 cm^−1^ of exposure time of styrene-ethylene-butadiene-styrene (SEBS).

The changes in FTIR spectra for the aged SEBS test pieces (Figure 1) clearly show the development of polar groups on the surface of the test pieces. These polar groups mainly consist of carboxyl, carbonyl, and hydroxyl groups [49,50,51].

Stretching and vibration is mainly seen in the regions corresponding to the hydroxyl groups (3800–3000 cm^−1^) and the carboxyl groups (1800–1600 cm^−1^), respectively, which indicates functional changes [52,53].

The vibration in the hydroxyl region shows the growth of a broad band between 3300 and 3500 cm^−1^, centered on 3430 cm^−1^, which corresponds to the hydroxyl groups associated with the carboxylic acids formed as a result of the ageing of SEBS [54,55].

The aliphatic hydroperoxides that also adsorb in the 3300–3350 cm^−1^ region are responsible for the formation of this broad band. Lastly, the regions between 3200 cm^−1^ and 3525 cm^−1^ can be attributed to the aromatic hydroperoxides and alcohols, respectively [56].

The changes observed in the 2000–1000 cm^−1^ region are the result of strong carbonyl absorbance observed in the range of 1750–965 cm^−1^. The presence of carboxylic groups is confirmed by the presence of peaks around 1650–1560 cm^−1^ that correspond to the anti-symmetrical deformation of the COO^−^ groups, and the peaks in the range of 1400–1310 cm^−1^ are synonymous with the formation of methyl ketones or aldehydes [57].

Ester absorption is observed at 1735 cm^−1^ and α,β-unsaturated in carbonyl species at 1680, 1660 and 1640 cm^−1^. In this case, only one peak was evident at 1660 cm^−1^. A small band also appeared at 1620 cm^−1^, and this could be ascribed to the vinyl groups or groups of conjugated vinyl [56,58]. The strong adsorptions below 1400 cm^−1^ are the result of the formation of methyl ketones or aldehydes, while the bands at 1135 and 1030 cm^−1^ are associated with a mixture of aliphatic esters and ethers, and that at 1135 cm^−1^ is more specifically ester –C–O–C– stretch [57].

Hydroperoxide build-up in the olefin phase is predominant, as shown by the FTIR analysis, and these species can give rise to crosslinking reactions of the type alkyl ± alkyl or alkyl ± peroxy radical recombinations. This will cause phase separation and eventual crosslinking of the olefin units to form carboxylic acids via aldehydes and then peracids (935 and 1770 cm^−1^) [10]. The increase in crosslinking is shown with the increase in the bands and with the UV exposure time of the samples.

Below 1000 cm^−1^, the adsorption bands are the result of species of vinyl di-substituted vinylidene at 880 cm^−1^ and tri-substituted vinyl at 845 cm^−1^. Peaks also appear in the 680–580 cm^−1^ region owing to the presence of hydroxyl groups resulting from the oxidation of the surface caused by the ageing of the test pieces [10].

### 3.2. Surface Morphology Study

The exposure of the samples to the light caused a superficial modification that was visible to the naked eye; for this reason, it was decided to perform a morphological study to evaluate the degradation with different microscopic techniques to be able observe the superficial modification at different magnifications, thus avoiding the loss of information when working at very high increases, as could happen in SEM and AFM techniques.

**Figure 2:**
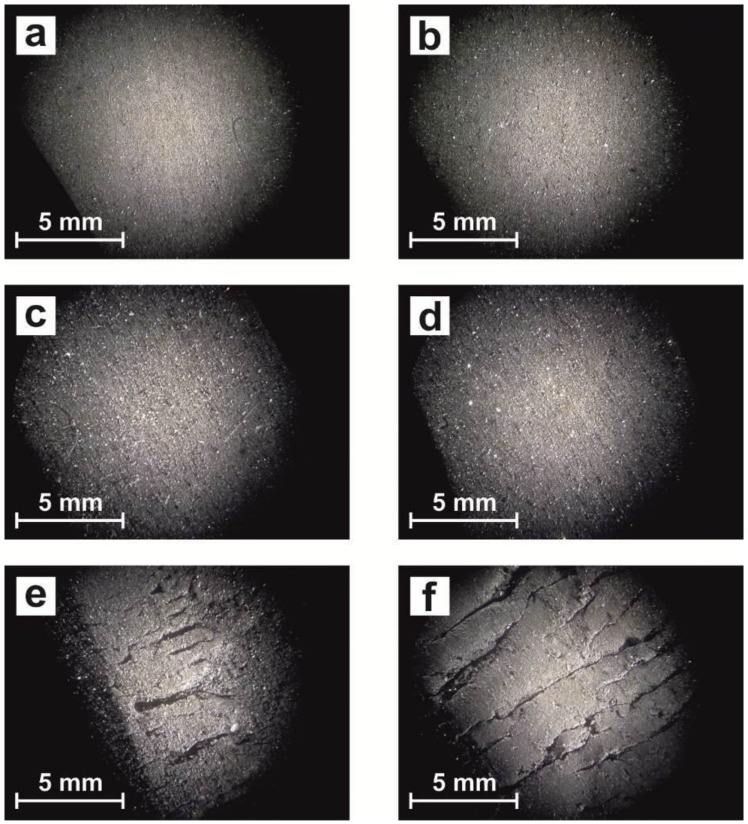
Stereoscopic loupe micrograph of surface of SEBS: (**a**) without exposure (T0), (**b**) UV exposure T1, (**c**) UV exposure T3, (**d**) UV exposure T5, (**e**) UV exposure T7, and (**f**) UV exposure T9.

For this reason, the exposure surface also was observed by stereoscopic loupe (Figure 2), where the effects of exposure clearly show an increase in the number and size of surface cracks when the exposure time increases. When SEBS was not exposed to light, the surface was completely smooth (Figure 2a) and, with low levels of exposure, the surface had a similar aspect (Figure 2b–d), but with high exposure to light (Figure 2e–f), the surface clearly changed and a lot of cracks appeared, which indicated the degradation process.

**Figure 3:**
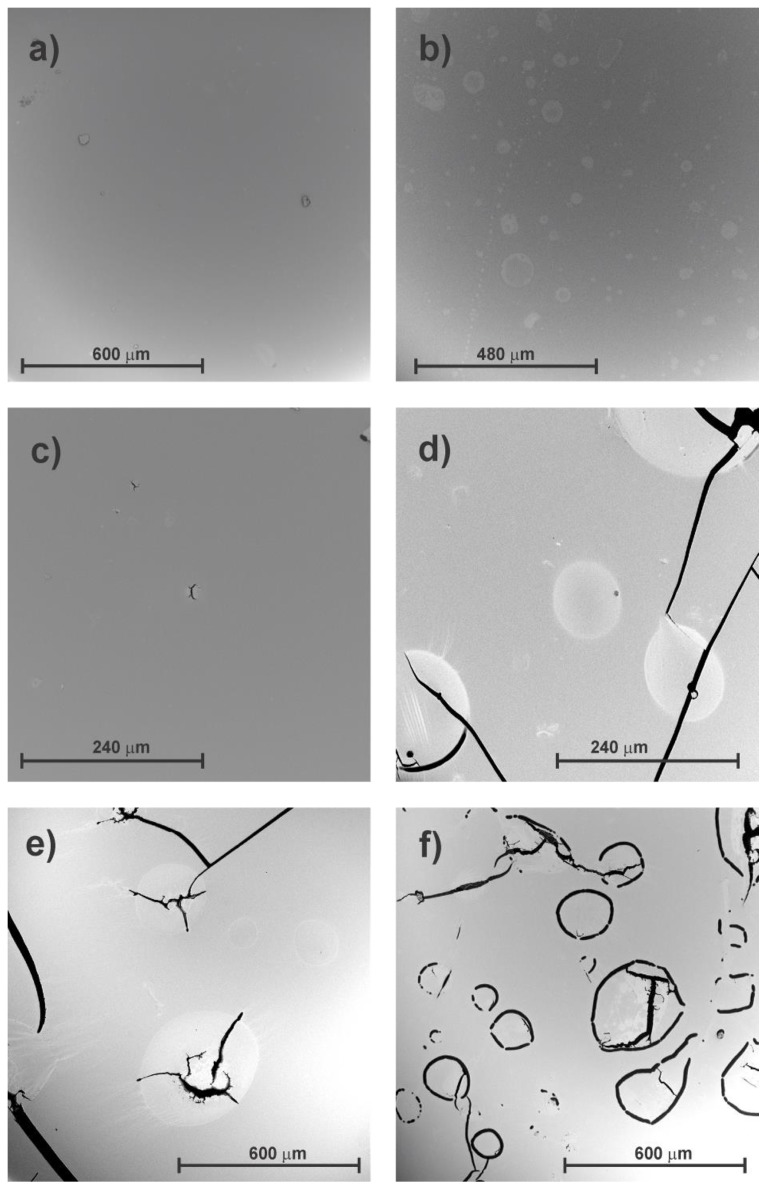
Scanning electron microscopy (SEM) micrograph of surface of SEBS: (**a**) without exposure (T0) (200×), (**b**) UV exposure T1 (250×), (**c**) UV exposure T3 (500×), (**d**) UV exposure T5 (500×), (**e**) UV exposure T7 (200×), and (**f**) UV exposure T9 (200×).

Scanning electron microscopy is a well-known electron-beam technique in which the dispersion of electrons is used to view the topography of sample surfaces. SEM has the potential to generate images with a resolution of a few nanometers, and it has a relatively wide depth of field [20]. The samples observed by SEM show an appearance similar to the samples observed by stereoscopic loupe, as the sample without exposure to UV light presents a smooth surface (Figure 3a). As exposure to light increases, however, spots and small cracks appear for shorter exposure times, T1 (Figure 3b) and T3 (Figure 3c). However, when exposure to UV light is higher, the number and size of cracks and spots on the surface of the material increase (Figure 3d–f), which indicates a clear symptom of material degradation.

AFM was used in order to obtain better information regarding the exposed surface of the SEBS test pieces. The areas on the exposed surface that do not show cracks were selected because an increase in the stickiness of the samples was observed in the samples as the exposure to UV light increased. AFM is also called a scanning probe microscope (SPM). This technique is able to continuously scan a surface topography by means of a probe or sharp pyramidal or conical point in order to generate topographical maps with a resolution in the vertical or horizontal plane of 0.01 nm [20]. This resolution allows the detection of changes in surface topography or other surface changes after test treatments through the measurement of roughness [55]. The roughness values obtained supplied valuable information regarding surface condition and adhesion force.

**Figure 4:**
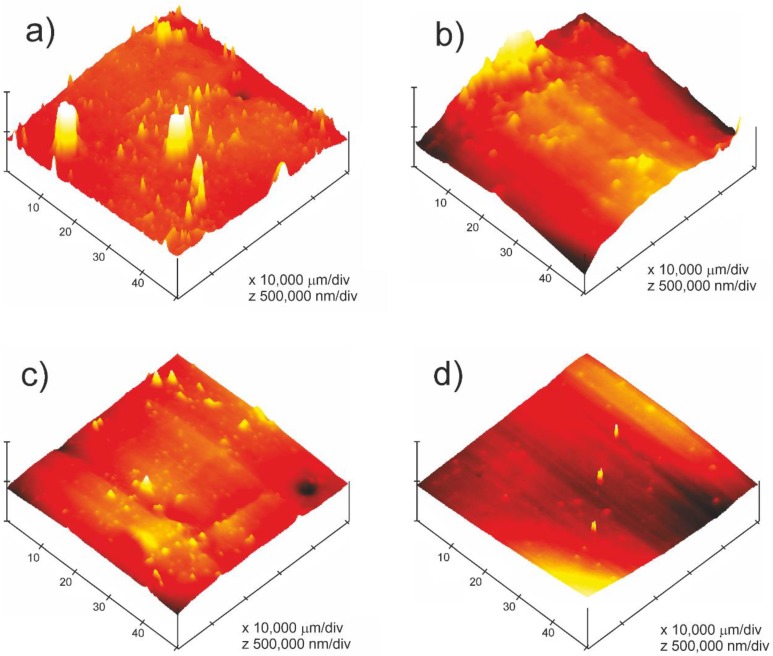
Atomic force microscopy (AFM) surface of SEBS: (**a**) without exposure (T0), (**b**) UV exposure T2, (**c**) UV exposure T7 and (**d**) UV exposure T9.

Figure 4 and Table 3 show how the surface roughness of SEBS decreases as the duration of UV exposure increases, from Rms values of 222 for the unexposed sample to 9.5 Rms for the sample exposed to UV light for longer (T9); this decrease is mainly a result of the degradation of the elastomeric phase. These results complement and confirm the loss of wettability results obtained by measuring contact angles, because the contact angle, in all of the liquids used, increases as the time of exposure to UV light of the samples increases and the surface energy of the samples decreases. Therefore, the degradation of SEBS caused by an exposure to UV light generates two surface effects. On the one hand, the formation of cracks, as well as a decrease in surface roughness in areas where there are no cracks. Both phenomena cause a loss of wettability of the material. The roughness values obtained for the surfaces analysed by AFM are shown in Table 3.

### 3.3. Mechanical Properties

**Figure 5:**
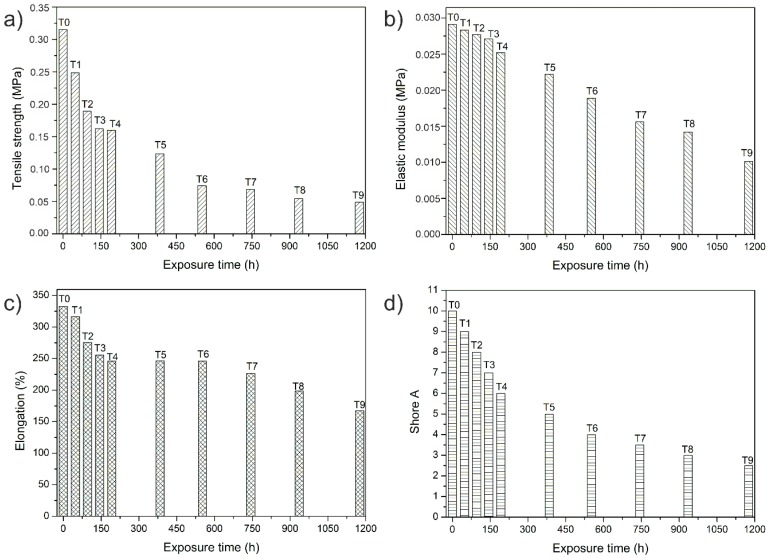
Mechanical properties versus UV exposures time of SEBS: (**a**) tensile strength, (**b**) elastic modulus, (**c**) elongation at break, and (**d**) hardness Shore A.

The mechanical properties of SEBS are also affected owing to UV exposure as a result of chain breakage and the formation of carboxylic groups, aliphatic esters, and ethers detected in the FTIR analysis. Figure 5a shows how the tensile strength decreases with an increase in UV exposure. The effect was most pronounced from T4 onwards when the resistance loss in relation to T0 reaches 50%. For T9, the resistance loss is close to 82% in relation to T0.

The elastic modulus behaves in a similar manner to the tensile strength (Figure 5b). There is a loss of rigidity in the material, although this is not as pronounced as the tensile strength. This is because of the fact that the material initially has an elastomeric nature. Exposure time T9 gives a loss of 64% in relation to T0.

Elongation at break (Figure 5c) also decreases with increased exposure to UV radiation, although, once again, this decrease is not as pronounced as for the tensile strength. The effects of UV exposure appear to occur from T4, and increase up to T9, producing a total decrease in elongation of 50% in relation to T0. This behavior is not as pronounced owing to the elastomeric characteristics conferred on the materials by its chemical structure.

Hardness values (Figure 5d) also show a decrease owing to the ageing caused by exposure to UV. There is an almost linear decrease in hardness in relation to exposure time. The greatest loss of hardness in relation to that shown without exposure is 75%.

Prolonged exposure to UV light causes a decrease in mechanical properties, both ductile and resistant properties, mainly owing to the functional groups that are generated during exposure to UV light and the cracks that were observed by SEM on the surface of the samples.. Furthermore, a rapid decrease in these properties was observed in relatively short time, that is, 192 h.

### 3.4. Analysis of Optical Contact Angle (OCA) and Surface Energy (γ_s_)

OCA is a surface-sensitive technique that allows measurement of wetting properties and the surface energy of a test piece. In general, polar and non-polar liquids are dispersed over the surface of the test piece and measurement is made of the angle that the liquid forms with the surface (as measured through the liquid). Smaller angles indicate a greater surface wetting and, therefore, a higher surface energy and work of adhesion [59,60,61,62]. As the surface energy and the wetting capacity are related to adhesion, the OCA provides an indirect measurement of adhesion, which allows comparison between the work of adhesion and the methods of direct bonding.

**Figure 6:**
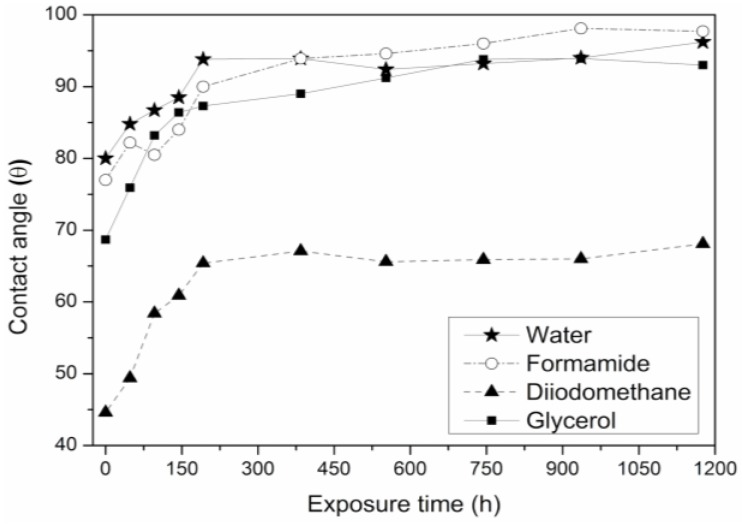
Variation of the contact angle (θ) on SEBS surface for different test liquids versus UV exposure time.

In the studied test pieces, the contact angle was initially low for samples with short exposure times. The contact angle then increased with exposure time and, therefore, wettability will be less, independent of the liquid used, Figure 6. This indicates that the morphology of the surface changed by exposure to UV radiation, as was observed on the surface roughness of the sample analyzed with AFM.

**Figure 7:**
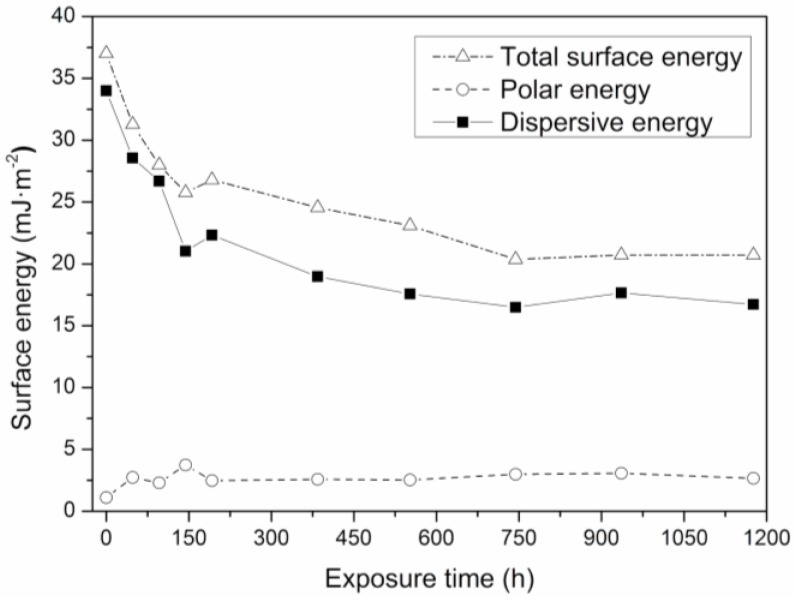
Variation of surface energy ($\gamma_{s}$) and its polar $\left(\gamma_{s}^{p}\right)$ and dispersive contributions $\left(\gamma_{s}^{d}\right)$ of the SEBS versus UV exposure time.

Total surface energy is seen to decrease as exposure time increases, Figure 7. Given the increase in contact angle (Figure 6), this indicates a change in the surface of the material and a reduced wettability [63,64]. All of this is related to the roughness of the surface, which is thought to decrease with exposure time as the surface energy decreases. The loss of surface energy in relation to the unexposed test piece (T0) is 54%.

### 3.5. Yellowness Index (YI)

**Figure 8:**
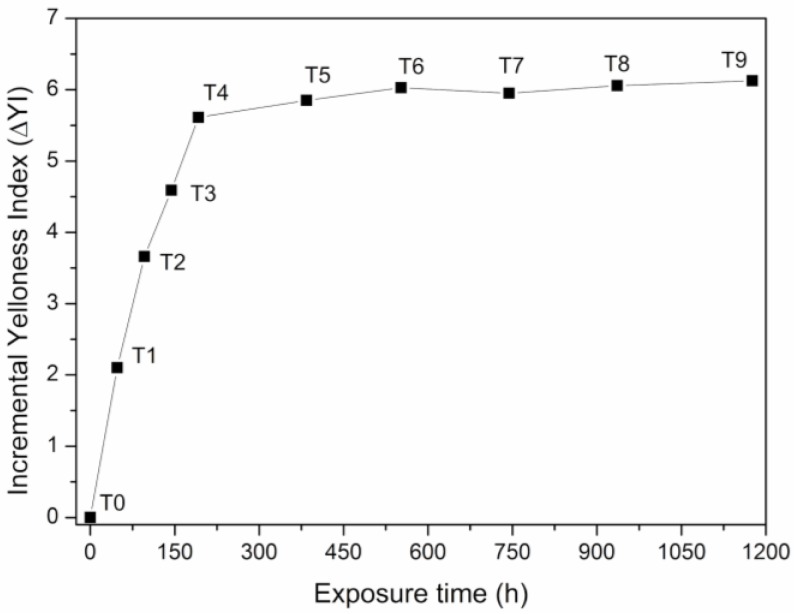
Incremental yellowness index (∆YI) versus UV exposure time of SEBS.

One of the most common problems in the manufacture and processing of polymers is the change of color shade owing to degradation processes. The colorimetric results for the SEBS test pieces show that there is an induction time for the formation of chromophores that absorb visible light [54]. However, after this initial yellowing, which is not visible to the naked eye, the YI values for SEBS reach a maximum value following lengthy UV exposure, that is, 192 h. From this exposure, yellowing increases, but very slightly, until the highest exposure time, as can be seen in Figure 8.

## 4. Conclusions

In the present work, the effect of UV exposure time on the chemical structure, as well as on the mechanical and morphological properties of SEBS, in order to test the behavior observed in the aged material was analyzed. As observed, the degradation of SEBS caused by accelerated aging results in structural changes to the material associated with the appearance of polar groups, mainly carboxyl, carbonyl, and hydroxyl groups. Morphologically, it is observed that, as the exposure time increases, the number and size of cracks and spots on the surface of the material increase, thus evidencing the degradation of the material. However, it is observed that the decrease in the roughness (Rms) of the surface as UV exposure increases is mainly a result of the degradation of the elastomeric phase, as more viscous surfaces are found in the more degraded test pieces as compared with the less degraded test pieces. Furthermore, it was observed how UV radiation gives rise to a yellowing of the exposed surface of the material (although this is not visible to the human eye), with said yellowing being greater as the exposure time increases. The degradation effect of UV exposure on SEBS samples on their mechanical properties was evidenced by a decrease in ductile and resistant properties as the UV exposure time increased, obtaining a reduction in tensile strength and elongation at break of 82% and 50%, respectively, for T9 with respect to the sample without degradation (T0). The effects of SEBS surface degradation were also evidenced by comparing surface energy results to those for unexposed material. As observed, the contact angles (θ) for droplets on the surface of the SEBS increase with UV exposure, evidencing a decrease in the wettability in the sample as the exposure time increases. This increase in contact angle (θ) involves the reduction of surface energy (γ_s_) with increasing UV exposure time, decreasing the surface energy by around 54% for the T9 sample with respect to the unexposed sample. Therefore, in the present work, it was shown how UV radiation negatively affects the properties of SEBS, leading to a decrease in its mechanical properties, increasing its yellowing and the appearance of surface cracks as the exposure time increases.

## Figures and Tables

**Table 1:** UV exposure times of samples of styrene-ethylene-butadiene-styrene (SEBS).

Samples	UV Exposure Time (Hours)
T0	0
T1	48
T2	96
T3	144
T4	192
T5	384
T6	552
T7	744
T8	936
T9	1176

**Table 2:** Characteristics of the different liquids used in the contact angle measurement for determining the energy surface samples of SEBS.

Liquid	γ_l_^d^ (mJ·m^−2^)	γ_l_^p^ (mJ·m^−2^)	γ_l_ (mJ·m^−2^)
Water	22.0	50.2	72.2
Glycerol	34.0	30.0	64.0
Diiodomethane	48.5	2.3	50.8
Formamide	32.3	26.0	58.3

**Table 3:** Roughness of UV exposure times of samples of SEBS.

Samples	Rms (nm)	Rmax. (nm)
T0	221.68	938.10
T1	195.36	825.69
T2	106.34	607.52
T3	78.29	589.64
T4	63.12	368.96
T5	46.31	254.24
T6	38.45	178.12
T7	20.21	102.23
T8	16.47	88.26
T9	9.49	74.34
